# Response of growth and development of the Pacific oyster (*Crassostrea gigas*) to thermal discharge from a nuclear power plant

**DOI:** 10.1186/s12898-018-0191-y

**Published:** 2018-09-06

**Authors:** Zhi-guo Dong, Yi-hua Chen, Hong-xing Ge, Xiao-ying Li, Hai-long Wu, Chen-he Wang, Zhe Hu, Yang-jian Wu, Guang-hui Fu, Ji-kun Lu, Hua Che

**Affiliations:** 1 Jiangsu Key Laboratory of Marine Bioresources and environment, Jiangsu Key Laboratory of Marine Biotechnology, Huaihai Institute of Technology, Lianyungang, 222005 Jiangsu China; 2 Co-Innovation Center of Jiangsu Marine Bioindustry Technology, Lianyungang, 222042 Jiangsu China; 3 Lianyungang City Marine and Fishery Development Promotion Center, Lianyungang, 222000 Jiangsu China; 4 Lianyungang Muyang Aquaculture Co., Ltd., Lianyungang, 222042 Jiangsu China

**Keywords:** Nuclear power plant, Thermal discharge, *Crassostrea gigas*, Growth and development

## Abstract

**Background:**

During electricity generation of nuclear power plant, heat energy cannot be completely converted into electrical energy, and a part of it is lost in the form of thermal discharge into the environment. The thermal discharge is harmful to flora and fauna leading to environmental deterioration, biological diversity decline, and even biological extinction.

**Results:**

The present study investigated the influence of thermal discharge from a nuclear power plant on the growth and development of Pacific oyster *Crassostrea gigas* which is widely used as bio indicator to monitor environmental changes. The growth of soft part and the gonad development of oysters were inhibited due to thermal discharge. During winter season, temperature elevation caused by thermal discharge promoted the growth of oyster shells. During summer season, the growth rate of oysters in thermal discharge area was significantly lower than that of the natural sea area.

**Conclusions:**

The results of this study provided a better understanding of assessing the impact of thermal discharge on the marine ecological environment and mariculture industry. It also provided a scientific basis for defining a safe zone for aquaculture in the vicinity of nuclear power plants.

## Background

China is one of the fastest growing countries in nuclear power industry in the world. So far, the number of nuclear power plants in China has reached to 34 and 20 nuclear power plants are being built, accounting for 40% of the total number of nuclear power plants in the world. China has a total installed nuclear power capacity of 53, 613 MW (e) as of July, 2017 [1]. However, an inherent problem in electricity generation is that the energy generated in the reactor cannot be converted into electrical energy [2]. Any reactor for that matter even a thermal power plant needs huge quantity of water for cooling the condenser and process water [3]. Considering the huge need of cooling water, nuclear power plants are mostly located in coastal areas. Nuclear power plants use seawater from the coastal area as a coolant for cooling the condenser by heat exchange. The warm water after extracting heat was released back into the sea directly [4, 5]. Thermal discharge as a source of pollution entering the sea increases the water temperature locally or regionally leading to thermal pollution [6].

Thermal pollution will not only increase the temperature of the seawater, but also change the physical and chemical properties. The rise in temperature will also lead to reduction in dissolved oxygen [7, 8] and increase the concentration of nitrogen and phosphorus in seawater [9]. The intensity and speed of temperature elevation at the sea surface can have a significant effect on biological growth, metabolism, reproduction and life cycle [10]. Researchers have found that temperature elevation can increase in the abundance of temperature-tolerant taxa [11], but it also reduces the abundance of temperature-sensitive species [12, 13]. Therefore, change in temperature is an important factor that results in decline of marine population and habitat destruction [14].

Changes in temperature will directly affect bivalves, because most of them are sessile, sedentary and immobile [15]. Moreover, in comparison to fish and other marine organisms, bivalves have a very low level enzyme system activity capable of metabolizing persistent pollutants [2]. Hence, bivalves are usually considered sensitive indicators of marine pollution [16–18]. Oysters are sessile and temperature sensitive bivalves. A considerable amount of research has been carried out to monitor the environment using oysters as an indicator [19–22].

China is one of the largest oyster farming countries in the world; in 2015, the total production of oysters exceeded 4573 million t, accounting for 33.7% of the total production of the total yield of shellfish [23]. Therefore, the high yield and quality of oysters is very important for the economic development of marine culture. However, the build-up of nuclear power plants, especially thermal discharge, has brought new challenges to the traditional farming model. Hence it is very important to research the growth and development of oysters under the influence of thermal discharge from nuclear power plants.

Tianwan Nuclear Power Plant (TWNPP), located on the coast of the Yellow Sea, has been running for 10 years since May 17, 2007 [24]. The reactor of TWNPP was VVER-1000/428 which was designed based on the Russian standard reactor type VVER-1000/320 [25]. Unit I was operated in 2007, and it had two pressurized water reactor, each of 1060 MW(e) capacity. Unit II will operate in December 2018, the capacity of pressurized water reactor will be 1100 MW(e). TWNPP operated with a cooling system and required 96.88 m^3^ s^−1^ of cooling water [26]. After extracting heat, the thermal discharge was subsequently released back into the sea. In this study, we carried out the experiments in three sampling stations in the vicinity of TWNPP up to a distance of 14.20 km on either side of the plant. The growth and development characteristics of oyster shells, meat yield (MY) and gonadosomatic index (GSI) were determined in order to assess the effect of thermal discharge from nuclear power plant on the growth and development of oysters. This will provide a better understanding of response of growth and development of the Pacific oyster to thermal discharge from a nuclear power plant.

## Methods

### Study area

**Fig. 1:**
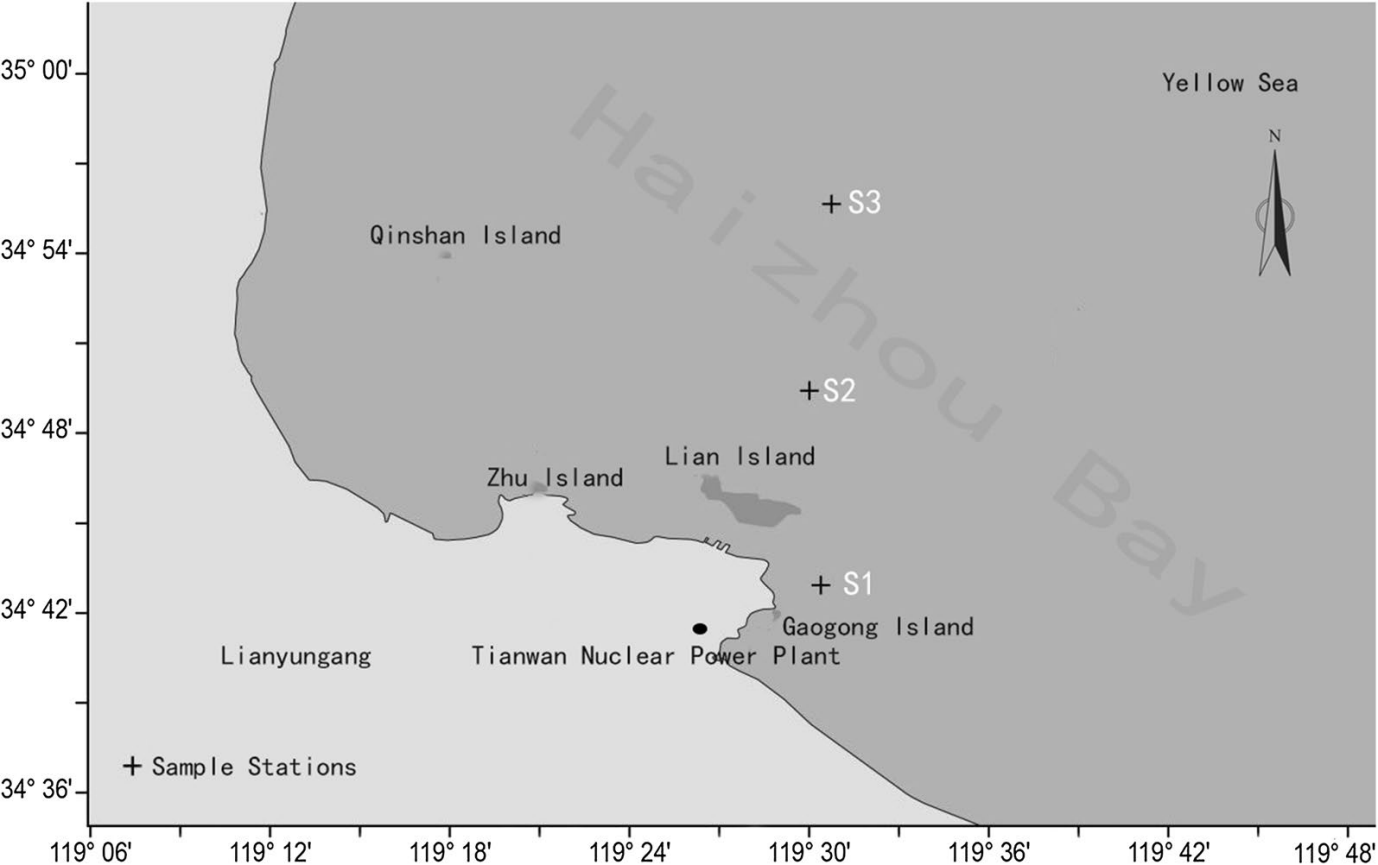
Study area and sample stations in Haizhou Bay

Haizhou Bay, which is adjacent to the TWNPP (34°41′ 13″ N, 119°27′ 53″ E), was selected as the study area. Three research stations, S1 (34°42′ 56″ N, 119°30′ 24″ E), S2 (34°49′25″ N, 119°30′ 07″ E) and S3 (34°55′ 39″ N, 119°30′ 45″ E), which are the principal oyster farming areas in Haizhou Bay were selected to carry out the present experiments (Fig. 1). S3 was taken as control area for it is far from the nuclear power plant (straight-line distance from the outfall 14.20 km) and the seawater temperature was not affected by thermal discharge. Chosen as the test area, S1 was closest to the outfall (4.92 km) and another station S2, 8.46 km away from the outfall also was selected in transition area.

The straight-line distance from the intake to the TWNPP is 5.21 km, and the outfall near the TWNPP. The temperature at the intake and outfall were showed in Table 1 from December 1, 2015 to April 30, 2016.

**Table 1:** The temperature at the intake and outfall of the TWNPP

	Dec-1, 2015	Dec-22, 2015	Jan-12, 2016	Feb-2, 2016	Feb-24, 2016	Mar-18, 2016	Apr-7, 2016	Apr-30, 2016
Intake temperature (°C)	11.2	8.9	7.2	5.3	6.2	7.6	13.4	21.8
Outfall temperature (°C)	73.4	72.2	70.6	66.2	69.8	71.2	73.6	75.2

### Oyster collection and data acquisition

Oysters were cultured by long line culture. We used scallop shells for holdfast, the seedling rope suspension was cultured in floating rope, and the length of rope for inserting seeding was 1.2 m. Oyster seeds were cultured at the sample stations on November 11, 2015. The initial gross weight was 12.50 ± 0.57 g, and the shell height (SH), shell length (SL) and shell width (SW) were 3.45 ± 0.38, 2.32 ± 0.37, and 1.15 ± 0.28 cm, respectively.

**Fig. 2:**
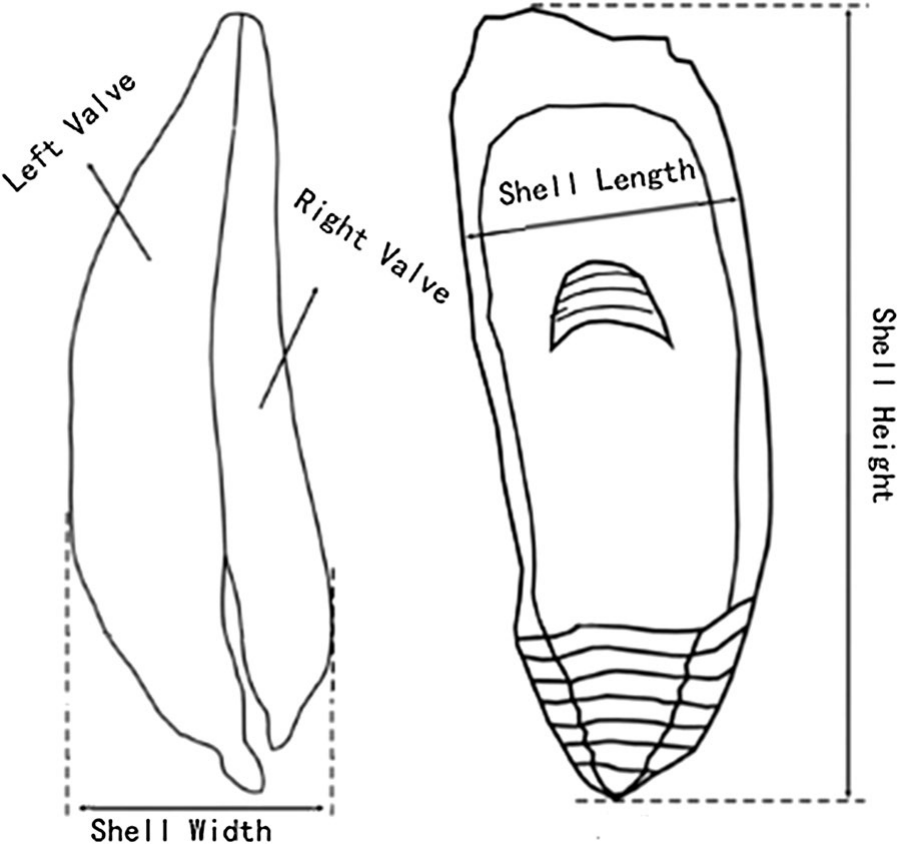
Diagram showing method of measuring the height, length, and width of oyster valves

50 samples of oyster were collected randomly at each sample station during the trial and were kept in foam boxes with ice packs. In the laboratory, the SH, SL and SW of oysters were measured using vernier caliper (accuracy of 0.02 mm,) according to published methods [27, 28] as shown in Fig. 2. In addition, we measured the gross weight, wet meat and gonad weight using electronic balance (accuracy of 0.0001 g) in order to calculate the MY and GSI. The formulas used to calculate the MY and GSI were as follows:

$$ {\text{MY}}\,\left( \%  \right) = \frac{{{\text{wet meat weight}}}}{{{\text{gross weight}}}} \times 100\%  $$

[29],

$$ {\text{GSI}}\,\left( \%  \right) = \frac{{{\text{gonad}}\,{\text{weight}}}}{{{\text{wet}}\,{\text{meat}}\,{\text{weight}}}} \times 100\% $$

[30].

### Measurement of environmental parameters

At the sample stations, the water temperature at 0.5 m below the surface was measured using a calibrated mercury thermometer (accuracy of 0.2 °C). And water samples for nutrients were collected from 10 to 15 cm below the sea surface with a 2 L water sampler. Water samples were stored in sampling bottle with a volume of 500 mL and kept on ice (about 4 °C) until measurements were made in lab. Concentrations of total nitrogen (TN) and total phosphorus (TP) were measured according to colorimetric methods (GB 17378.4-2007).

Water samples for phytoplankton were collected by vertical trawl from the bottom to the sea surface with a plankton net (0.37 m diameter, 0.1 m^2^ mesh area, 77 μm mesh size) [31]. After collection, samples were preserved in 5% formaldehyde immediately. Phytoplankton were observed and counted on a scaled slide (0.1 mL) using a light microscope [32, 33].

### Statistical analysis

All statistical analyses were conducted using SPSS software (v.17.0 SPSS Inc.). Comparisons between groups were tested by one-way analysis of variance (One-Way ANOVA) and Duncan test. The result was expressed as mean ± SD, differences were considered to be significant at *P *< 0.05.

## Results

### Variation of temperature at sample stations

**Fig. 3:**
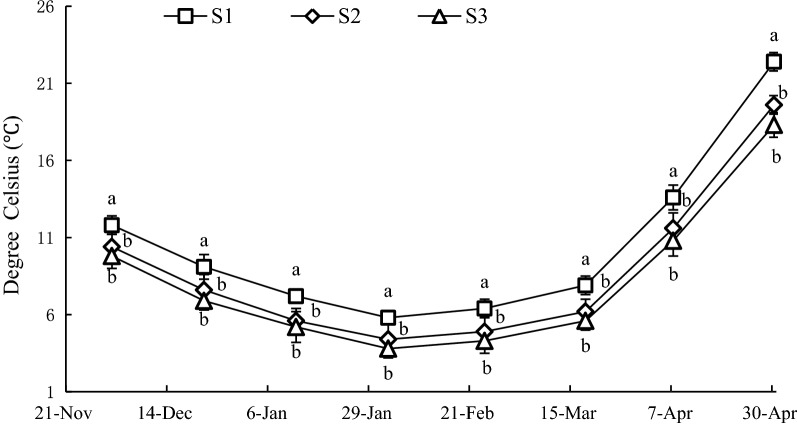
Variation of temperature at sample stations. Different letters of the same sampling date indicate significant difference at 0.05

Temperature at S1, S2 and S3 were in the following ranges: 5.8–22.4, 4.4–19.6, and 3.8–18.3 °C, respectively. The mean temperatures at S1, S2 and S3 were 10.5 ± 5.5, 8.8 ± 5.1 and 8.1 ± 4.8 °C, respectively. The temperature at S1 was significantly higher (*P *< 0.05) than that of S2 and S3 during the whole experiment (Fig. 3), however, there were no significant differences (*P *> 0.05) between S2 and S3.

### Growth of *C. gigas* shells

#### Variation of SH

**Fig. 4:**
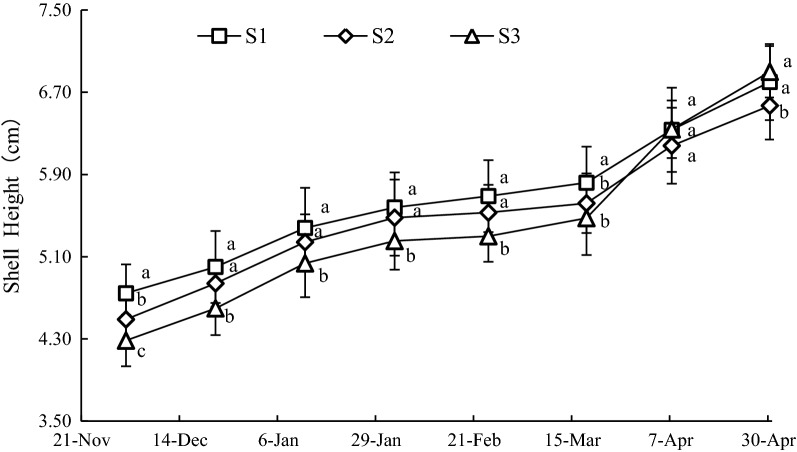
Variation of SH at sample stations. Different letters of the same sampling date indicate significant difference at 0.05

The growth of SH was consistent over the three sample stations (Fig. 4). The mean values of SH at S1, S2 and S3 were 5.67 ± 0.72, 5.48 ± 0.72 and 5.41 ± 0.93 cm, respectively. The SH at S1 was significantly larger (*P *< 0.05) than that of S3 before March 18, 2016. Significant increment of SH was observed among the three sample stations after March 18, 2016. And the growth of SH at S3 was the greatest. On April 7, 2016, the SH at S3 had exceeded the others, and there were no significant differences (*P *> 0.05) among the three sample stations.

#### Variation of SL

**Fig. 5:**
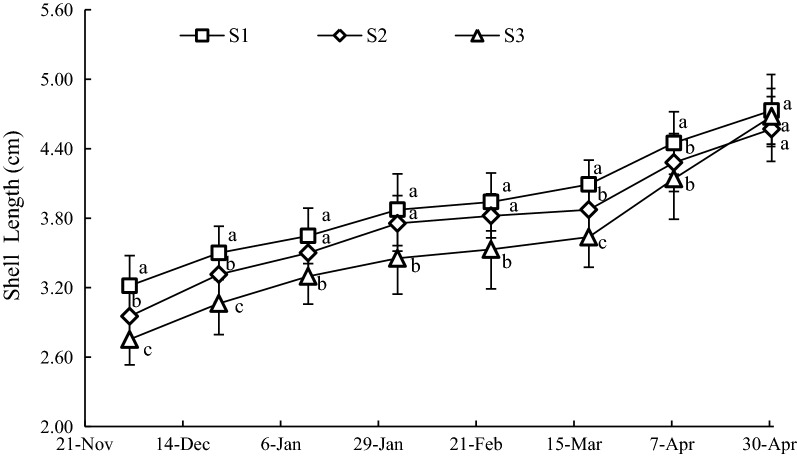
Variation of SL at sample stations. Different letters of the same sampling date indicate significant difference at 0.05

The growth of SL at all sample station showed similar patterns as SH (Fig. 5). The mean values of SL at S1, S2 and S3 were 3.93 ± 0.53, 3.74 ± 0.56 and 3.57 ± 0.65 cm, respectively. Before March 18, 2016, the SL showed continuous growth, and the SL at S1 was significantly larger (*P *< 0.05) than that of S3. After March 18, 2016, significant increment of SL was observed at the three sample stations. The SL at S3 was still the smallest on April 7, 2016, however, it exceeded the SL at S2 on April 30, 2016 (*P *> 0.05), and it was still lower than that at S1 (*P *> 0.05).

#### Variation of SW

**Fig. 6:**
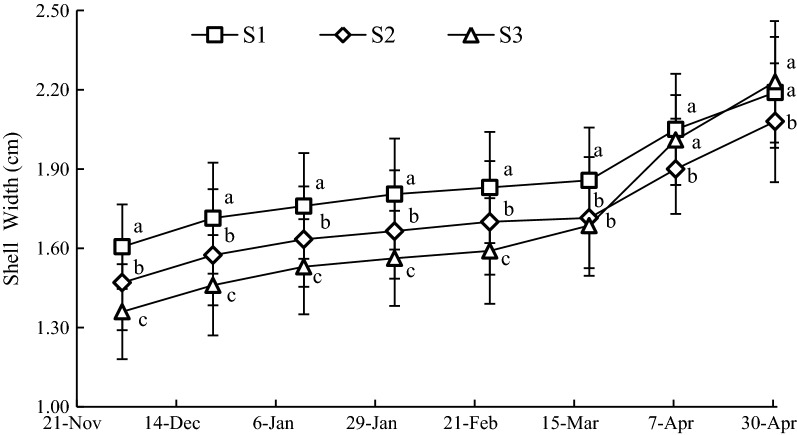
Variation of SW at sample stations. Different letters of the same sampling date indicate significant difference at 0.05

The growth of SW was showed in Fig. 6, the mean values of SW at S1, S2 and S3 were 1.85 ± 0.20, 1.72 ± 0.21 and 1.67 ± 0.32 cm, respectively. The maximum of SW was observed in S1, followed by S2 and S3 on February 24, 2016, and there were significant differences (*P *< 0.05) among the three sample stations. However, the SW at S3 exceeded S2 on April 7, 2016 (*P *< 0.05), and it was still significantly lower than S1 (*P *> 0.05). On April 30, 2016, the SW of S3 was higher than that of S1, however, there were no significant differences between the two (*P *> 0.05).

### Variation of MY at the three sample stations

**Fig. 7:**
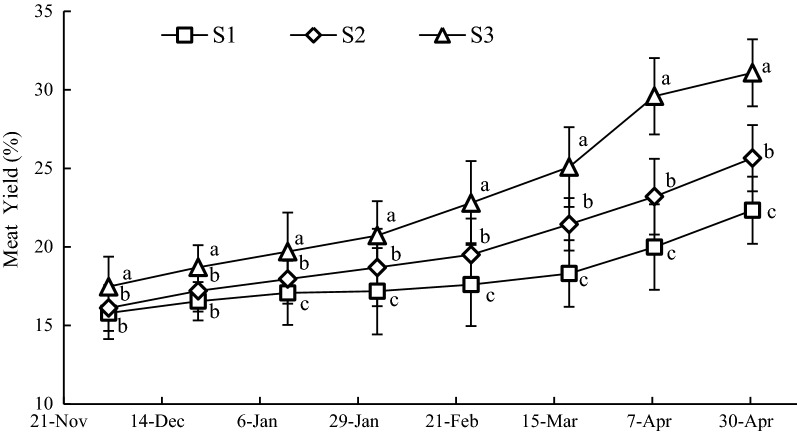
Variation of MY at sample stations. Different letters of the same sampling date indicate significant difference at 0.05

The MY of oysters at S3 was the highest over the duration of the experiment, followed by S2 and S1 (Fig. 7). The mean values of MY at S1, S2 and S3 were 18.42 ± 2.71%, 20.53 ± 3.72%, and 23.12 ± 5.45%, respectively. The MY at S3 was significantly greater (*P *< 0.05) than that of S1 and S2 before November 22, 2015, however, there were no significant differences (*P *> 0.05) between S1 and S2 at the same time. After November 22, 2015, there were significant differences (*P *< 0.05) among the three sample stations.

### Variation of GSI at the three sample stations

**Fig. 8:**
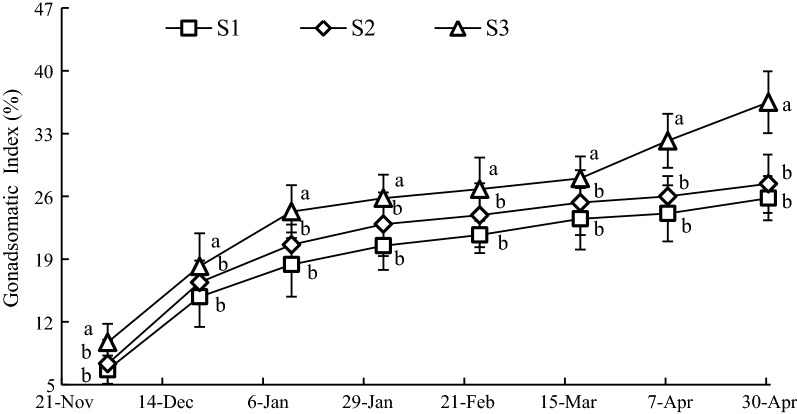
Variation of GSI at sample stations. Different letters of the same sampling date indicate significant difference at 0.05

The GSI of oysters at S3 was the highest for the duration of the experiment, followed by S2 and S1 (Fig. 8). The mean values of GSI at S1, S2 and S3 were 18.84 ± 6.72%, 21.32 ± 6.39% and 24.93 ± 8.88%, respectively. Throughout the experiment, the GSI at S3 was significantly greater (*P *< 0.05) than that of S1 and S2, however, there were no significant differences (*P *> 0.05) between the S1 and S2.

### Variation in phytoplankton density at the three sample stations

**Fig. 9:**
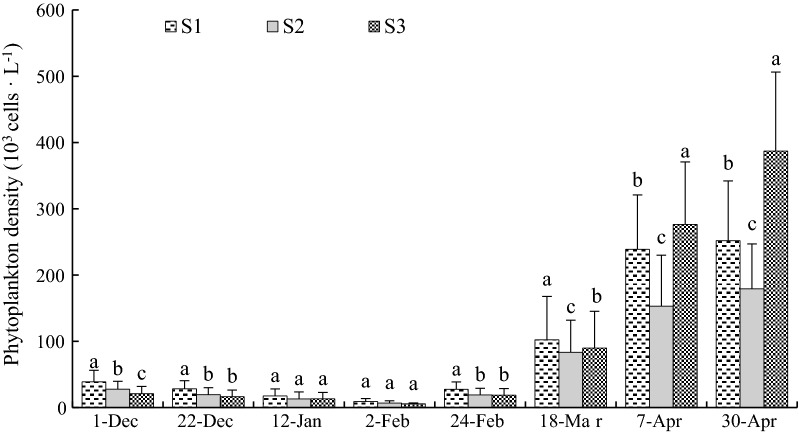
Variation in phytoplankton density at sample stations. Different letters of the same sampling date indicate significant difference at 0.05

During the trial, the phytoplankton density decreased at first and then increased (Fig. 9). The phytoplankton density at S1 was the highest from December 1, 2015 to February 24, 2016, followed by S2 and S3. After February 24, 2016, the phytoplankton density rose sharply, and there were significant differences (*P *< 0.05) among the three sample stations. After March 18, 2016, the phytoplankton density at S3 was the highest, followed by S1 and S2.

## Discussion

It is difficult to convert thermal energy into electric energy completely during the operation of a nuclear power plant; part of the thermal energy enters the receiving water in the form of thermal discharge, which directly results in a raised seawater temperature. Research has shown that the temperature is over 4 °C higher than the receiving water and it may cause thermal pollution if the thermal discharge into the ocean occurs for a long time [34]. Normally, thermal discharge from power generating plants is roughly 10 °C warmer than receiving waters [35]. S1 is closest to the nuclear power plant outfall, and the seawater is most affected. In our study, S1 had the highest water temperature over the duration of the experiment, and the mean value of the temperature was 2 °C higher than that of other sample stations. The mean values of temperature in S1, S2 and S3 was 10.5, 8.8, 8.1 °C, respectively, and this illustrates that the temperature elevation decreases as the distance from the outlet increases, which is in accordance with previous work Mei et al. [36].

Oysters are very sensitive to environmental change, and temperature variation greatly affects oyster growth. Researchers have shown that the growth of oyster shells is closely related to the source of food and the growth environment temperature; moreover, temperature elevation is beneficial to the growth of oyster shells in a suitable temperature range [37]. The present study showed that the SH of oysters cultured at S1 was greater than that of S3 from December 1, 2015 to March 18, 2016. We also found that the temperature at S1 was higher than at S3. The value of SL and SW showed a similar pattern at the same time points. These results indicate that the temperature elevation caused by thermal drainage promotes shell growth of oysters in low temperature season. The effect of thermal discharge is greater in winter than in summer [38, 39], which means that the lower the natural seawater temperature, the greater the effect of thermal discharge on seawater temperature. Studies have shown that it is favorable for growing oysters when the temperature elevation is below 4 °C [40, 41], and this may be the reason for the SH, SL and SW of oysters in sample S1 being greater than the other two sample stations before March 18, 2016.

**Fig. 10:**
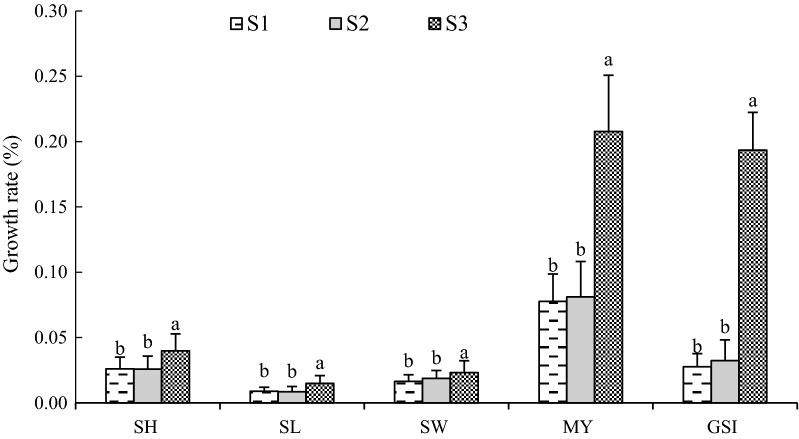
The growth rate of oyster after March 18, 2016. Different letters of the same index indicate significant difference at 0.05

The most important factor affecting reproduction is temperature [42, 43], and the gonadal development of marine bivalves have a close relationship with the fluctuation in the water temperature of their habitat [44]. The metabolism of bivalves is vigorous in the suitable temperature environment, and temperature elevation has positive effects on feeding, growth, gonadal development and reproduction [45]. The present study, however, leads to the opposite result. For instance, the S3 sample station, with the lowest temperature, had the highest GSI and MY during the trial. The GSI and MY of oysters at S1, with the highest temperature, was lower than that of S3. These results indicate that the temperature elevation caused by thermal discharge did not exert a positive effect on the gonadal and soft parts development of oysters, but conversely had an inhibitory effect. Especially after March 18, 2016, the GSI and MY showed significant increase at S3, however, the increment at S1 and S2 was not obvious. It has been shown that the effect of thermal discharge depends on ambient temperature regime [3]. Some marine animals may have already lived closer to the upper limit of lethal temperature and any further increased was more likely to affect them adversely, such as tropical region [46]. Researchers have showed that as soon as the lethal temperature is reached, the bivalves will die [2]. After March 18, 2016, temperature rise due to different seasons, the effect of thermal discharge was more obvious. Therefore, this may be the reason that GSI and MY at S1and S2 were lower than that of S3. We also found that the growth rate of GSI and MY at S3 was significantly higher than that at S2 and S1 (Fig. 10). This may result from that the temperature increment may not promote the growth of gonadal and soft parts of oysters due to the long-term influence of thermal discharge in high temperature season. However, the development of gonads depends not only on the water temperature but also on the accumulation of nutrients, and the accumulation of nutrients has a close relationship with bait quality and quantity. Therefore, the gonadal development of oysters is also influenced by the combination of bait, water environment and phytoplankton density [47, 48].

Temperature increment is easy to cause marine pollution, such as dissolved oxygen reduced [49], non-ionic ammonia increased [50], and even leads to the red tide [51, 52]. Oysters are filter feeders, and their gills are constantly exposed to the outside environment for filtration and respiration. The total ATPase activities in gill tissues will be reduced due to exposure of the polluted effluent over a long period of time [19, 53]. This will reduce the filtration rate of the gills, while preventing the most direct energy conversion in the body [54]. Moreover, environmental pollution can reduce the growth rate of organisms, because the pressure of environmental pollution can lead to organisms use energy to detoxify the body, which also depletes the energy reserves of the oyster [55, 56]. It may also be the reason that the growth and development of GSI and MY are inhibited: on the one hand, it may be that temperature elevation has an inhibitory effect on the growth of the oyster, on the other hand, the change of other environmental factors that may be caused by thermal discharge affects the growth of oysters adversely.

Phytoplanktons are the base of aquatic food web and the most important source of food for bivalves. Temperature is a direct factor that affects the growth of phytoplankton [57], particularly in higher temperatures areas [58], and temperature elevation increases the abundance of temperature-tolerant taxa [59]. According to the study of Jiang et al. [60], the thermal discharge from Ninghai Power Plant increased the phytoplankton abundance in Xiangshan Bay. The present study was consistent with that conclusion, as the phytoplankton density at S1 was the highest from December 1, 2015 to March 18, 2016 (Fig. 9), and this trend seemed to be closely related to an increase in nutrients at S1 (Table 2). TP and TN are inorganic nutrients that can limit the phytoplankton production in tropical marine ecosystems [61]. The higher concentration of TP and TN may be increased the phytoplankton abundance. Therefore, the higher phytoplankton density may be the reason for the elevated growth of SH, SL and SW at S1. Nevertheless, once the temperature of seawater rises beyond the optimum living temperature of aquatic organisms, it will adversely affect the survival, growth and reproduction of aquatic organisms [62]. This may explain why the phytoplankton density at S1 was lower than that of S3 after March 18, 2016. There was a significant growth of oyster shell at all sample stations after March 18, 2016, according to Moullac et al. [63], pearl oyster growth was correlated with Chl *a* resources, which corresponded to food supply. In this study, the phytoplankton density rose sharply after February 24, 2016. Therefore, the increase of food and food categories maybe an important reason for the promotion of growth and development of oysters after March 18, 2016.

**Table 2:** Concentrations of total phosphorus (TP) and total nitrogen (TN) at sample stations

Sample stations	TP (mg/L)	TN (mg/L)
S1	0.15 ± 0.02^a^	0.57 ± 0.04^a^
S2	0.16 ± 0.08^a^	0.53 ± 0.08^a^
S3	0.13 ± 0.02^b^	0.48 ± 0.02^b^

Different letters of the same column indicate significant difference at 0.05

## Conclusion

The present study delineated that thermal discharge from the nuclear power plant could affect the seawater temperature, nutrients, and phytoplankton density, especially the growth and development of the oysters. Temperature elevation caused by thermal discharge promotes the growth of oyster shells. Nevertheless, the growth of soft parts and gonad development are inhibited. The temperature increment may not promote the growth of gonadal and soft parts of oysters due to the long-term influence of thermal discharge. In consideration of the above muddling inferences, in order to optimize the development between mollusk culture and nuclear power, mollusk farming is not recommended near to nuclear power stations.

## Abbreviations

TWNPP: Tianwan nuclear power plant
MY: meat yield
GSI: gonadosomatic index
SH: shell height
SL: shell length
SW: shell width
TN: total nitrogen
TP: total phosphorus
